# C-953-01. Automating Burn Surgery Billing: A Large Language Model Pipeline for Operative Note CPT Code Extraction

**DOI:** 10.1093/jbcr/irag033.125

**Published:** 2026-04-06

**Authors:** Brendan T Fox, Brian R Quaranto, Jarrett Santorelli, Dylan Tanzer, Marcy B Jordan, William Flynn, James Lukan, Michael Chopko

**Affiliations:** University at Buffalo Jacobs School of Medicine and Biomedical Sciences, Buffalo, NY; University at Buffalo Jacobs School of Medicine and Biomedical Sciences, Department of Surgery, Buffalo, NY; University of San Diego, San Diego, CA; University at Buffalo Jacobs School of Medicine and Biomedical Sciences, Buffalo, NY; University at Buffalo, The State University of New York, Buffalo, NY; University at Buffalo Jacobs School of Medicine and Biomedical Sciences, Buffalo, NY; University at Buffalo, The State University of New York, Buffalo, NY; University at Buffalo Jacobs School of Medicine and Biomedical Sciences, Buffalo, NY

## Abstract

**Introduction:**

Burn surgery involves complex procedural combinations that challenge accurate medical billing. Surgeons must navigate intricate CPT coding rules specific to burn care, including total body surface area calculations, graft types, and concurrent procedures. Manual coding is time-intensive and error-prone, potentially leading to revenue loss and compliance issues. This study demonstrates the feasibility and preliminary results from an automated large language model (LLM) pipeline for extraction of CPT codes from burn operative notes.

**Methods:**

We developed a six-stage processing pipeline combining large language models with rule-based validation (Fig. 1). Complete operative notes from burn procedures were processed through: (1) entity extraction using OpenAI GPT-5 to identify procedural descriptions, (2) candidate CPT code retrieval using fuzzy matching against an annotated CPT database, (3) code confirmation through GPT-5 enhanced with burn-specific billing rules, (4) strict JSON enforcement for structured output, and (5) delivery through a web-based interface. A sample of n = 40 full-text operative notes from burn surgeries performed at our regional burn treatment center were abstracted from the clinical record and selected for analysis by the LLM pipeline.

**Results:**

The LLM pipeline successfully processed each record, assigning a mean of 7.33 ± 4.99 CPT codes per case with 20.85 ± 25.27 add-on CPT instances, demonstrating significant procedural complexity variation. Fig. 2 visualizes this case complexity variation by plotting the LLM-predicted primary codes on the x-axis and the add-on instance codes (i.e., CPT codes for each additional 100 cm2) on the y-axis, finding 27.5% low complexity (≤5 add-on codes) cases, 37.5% medium complexity (6-20 add-on codes) cases, and 35.0% high complexity (>20 add-on codes) cases.

**Conclusions:**

This automated pipeline successfully extracts CPT codes from unstructured burn operative notes by combining the contextual understanding of large language models with the precision of rule-based validation specific to burn billing requirements. The multi-stage approach ensures both comprehensive code identification and adherence to complex burn surgery billing guidelines. We are improving the pipeline in collaboration with board-certified burn surgeons, who are reviewing and validating the accuracy of LLM-predicted CPT codes.

**Applicability of Research to Practice:**

This system offers immediate practical value for burn centers by reducing coding time, improving billing accuracy, and allowing surgeons to focus on clinical care rather than administrative tasks. The pipeline could be integrated into existing electronic health records and billing systems, providing real-time coding suggestions that can be reviewed and confirmed by billing specialists.

**Funding for the Study:**

N/A.

